# Associations between emergency call stroke triage and pre-hospital delay, primary hospital admission, and acute reperfusion treatment among early comers with acute ischemic stroke

**DOI:** 10.1007/s11739-023-03349-x

**Published:** 2023-06-27

**Authors:** Martin F. Gude, Jan B. Valentin, Helle C. Christensen, Søren Mikkelsen, Morten B. Søvsø, Grethe Andersen, Hans Kirkegaard, Søren P. Johnsen

**Affiliations:** 1 Research and Development, Prehospital Emergency Medical Services, Central Denmark Region and Aarhus University, Aarhus, Denmark; 2 Danish Center for Clinical Health Services Research, Department of Clinical Medicine, Aalborg University, Aalborg, Denmark; 3 Copenhagen Emergency Medical Services, Capital Region of Denmark, Copenhagen, Denmark; 4 The Prehospital Research Unit, Region of Southern Denmark, Odense University Hospital, Odense, Denmark; 5 Centre for Prehospital and Emergency Research, Aalborg University and Aalborg University Hospital, Aalborg, Denmark; 6 Department of Neurology, Aarhus University Hospital, Aarhus, Denmark; 7 Department of Clinical Medicine, Aarhus University, Aarhus, Denmark; 8 Danish Clinical Quality Program (RKKP), National Clinical Registries, Copenhagen, Denmark; 9 Department of Anaesthesiology and Intensive Care, Aarhus University Hospital, Central Denmark Region, Aarhus, Denmark

**Keywords:** Ischemic stroke, Emergency medical services, Thrombolytic therapy, Time-to-treatment

## Abstract

To investigate the association between the Emergency Medical Service dispatcher’s initial stroke triage and prehospital stroke management, primary admission to hospitals offering revascularization treatment, prehospital time delay, and rate of acute revascularization. In an observational cohort study, patients with acute ischemic stroke (AIS) in Denmark (2017–2018) were included if the emergency call to the Emergency Medical Dispatch Center (EMDC) was made within three hours after symptom onset. Among 3546 included AIS patients, the EMS dispatcher identified 74.6% (95% confidence interval (CI) 73.1–76.0) correctly as stroke. EMS dispatcher stroke recognition was associated with a higher rate of primary admission to a hospital offering revascularization treatment (85.8 versus 74.5%); producing an adjusted risk difference (RD) of 11.1% (95% CI 7.8; 14.3) and a higher rate of revascularization treatment (49.6 versus 41.6%) with an adjusted RD of 8.4% (95% CI 4.6; 12.2). We adjusted for sex, age, previous stroke or transient ischemic attack, and stroke severity. EMDC stroke recognition was associated with shorter prehospital delay. For all AIS patients, the adjusted difference was − 33.2 min (95% CI − 44.4; − 22.0). Among patients receiving acute revascularization treatment (*n* = 1687), the adjusted difference was -12.6 min (95% CI − 18.9; − 6.3). Stroke recognition by the EMS dispatcher was associated with a higher probability of primary admission to a hospital offering acute stroke treatment, and subsequently with a higher rate of acute revascularization treatment, and with an overall reduction in prehospital delay.

## Background

Stroke is a time-critical condition requiring urgent treatment due to time-related, irreversible loss of brain cells and subsequent loss of physical and cognitive functions and increased mortality [1, 2]. Hence, a shorter treatment delay directly improves clinical outcomes [3–12], and the effect of reducing treatment delay may be observed continuously as the delay diminishes [13–16].

Activation of the Emergency Medical Service (EMS) by an emergency call to the Emergency Medical Dispatch Center (EMDC) is associated with a reduced prehospital delay, earlier emergency department (ED) arrival, and faster treatment than self-admission, referral from other medical facilities, and admission organized by general practitioners [17–22]. In the EMS system, a stroke may be identified either by the EMS dispatcher at the EMDC during the emergency call or by the EMS provider attending the patient on scene. The EMS dispatchers’ stroke identification directly affects treatment delay because the dispatchers determine the urgency level set for ambulances. A high urgency level to individuals with suspected stroke is associated with a reduced transport time [23] and an increased rate of thrombolysis compared with less urgent responses [24]. Patients whom the EMS dispatcher suspects suffer from a stroke also have a higher probability of being assessed prehospitally with a stroke screening tool than patients not suspected of stroke [25].

EMS dispatcher stroke recognition rates vary and are generally low with sensitivities ranging from 37 to 83%, and positive predictive values fall in the 30–68% range [26–30]. In Denmark, one-third of all stroke cases have been shown to be unrecognized by EMS dispatchers [27] which equals the performance in other countries [25, 31–33]. The use of a symptom-based stroke scale by an EMDC is associated with higher recognition of stroke/TIA [27, 33–35]. The use of a stroke assessment tool by the EMS dispatchers is recommended in the 2019 guideline from the American Stroke Association [36].

Studies focusing on EMS dispatcher stroke identification among potential candidates for acute revascularizing treatment are few [26, 33]. Associations between EMDC stroke triage and stroke management have not previously been isolated to a stroke subtype where acute treatment is well established or to a group of patients who from a timely perspective would all be considered candidates for acute stroke treatment at the time EMDC contact [26].

We aimed to evaluate the association between EMDC stroke triage and prehospital stroke management in patients with acute ischemic stroke (AIS) who contacted the EMDC within three hours of symptom debut. We compared patients with recognized stroke and patients with unrecognized stroke (EMDC stroke triage) with regards to primary admission to hospitals offering revascularization treatment, prehospital delay (time from emergency call to arrival at hospital), and the use of intravenous (i.v.) thrombolysis and/or endovascular treatment (EVT).

## Methods

### Study design and setting

This was a national observational cohort study including stroke patients (AIS) from all five health regions in Denmark. Patients with a final stroke diagnosis (AIS) were retrospectively identified in data from Danish healthcare registers in 2017 and 2018.

#### The emergency medical service (EMS)

In Denmark, healthcare including prehospital care is tax-financed and free for all citizens. Denmark is divided into five regions; each governed one EMDC and ambulance services [37]. In the study period, the ambulance services were managed partly by private contractors and partly by the regions. Extensive quality requirements for all ambulance services were described and controlled by the EMS authority of each region.

#### Ambulance personnel

All ambulances were manned with two EMS providers, counting at least one paramedic or an emergency medical technician intermediate (EMT-I). EMS providers did not use a symptom-based stroke assessment tool except in one region where a modified Cincinnati Prehospital Stroke Scale was being implemented during 2018 as part of a clinical study.

#### Stroke care system

In Denmark, treatment with i.v. thrombolysis has been centralized, producing an improved overall quality of care [38]. Hence, i.v. thrombolysis was not offered at all hospitals with an ED. In the study period, 11 hospitals offered acute stroke admission including i.v. thrombolysis. Among these 11 hospitals, three did not have a general ED, and acute admission was offered only to patients with suspicion of stroke. Furthermore, 14 hospitals had an ED but no acute stroke admission.

When stroke was suspected, either guided by the prenotification from the EMDC or by the patient’s clinical presentation, a telephone conference call from the EMS provider to a stroke neurologist was conducted; and it was decided whether the patient would be admitted directly to a stroke center or a general ED. Patients without suspicion of stroke would be admitted at the nearest ED. Because of the organization of the Danish hospitals, this nearest ED would not necessarily be located at a hospital with a stroke center with the capacity to initiate acute revascularization treatment. Hence, patients for whom a stroke was suspected, could experience a slightly longer prehospital transport to ensure a direct admission to nearest stroke center to offer immediate stroke diagnostics and treatment [39]. In the study period, EVT was offered at four Comprehensive Stroke Centers, with the relative timeline for most treatments of six hours. All Comprehensive Stroke Centers also performed i.v. thrombolysis.

#### Criteria-based dispatch reference work used in the EMDC and stroke triage

The EMDC was operated by EMS dispatchers who dispatched all emergency responses. Dispatch or triage was guided by criteria-based dispatch reference work (Danish Index) divided into main groups of symptoms (e.g., trauma, blocked airway, bleeding, burns, chest pain, etc.) specifying the level of urgency (A–E) [40]. Symptoms related to stroke and transient ischemic attack (TIA) produced a positive EMDC stroke triage. Symptoms of stroke triggered the most urgent level of response (level A); stroke symptoms that were considered to have resolved completely, interpreted as TIA, triggered a less urgent response level (level B) [41].

Level-A responses were dispatched within minutes; level-B responses, up to 30 min from the time of triage; level-C responses, within 1 or 2 h (C60 or C120); level D or E responses, within a varying period and when no treatment was needed. If a stroke was missed by the EMS dispatcher, the emergency response was dispatched from a non-stroke-associated symptom group (describing a non-stroke symptom) in the Danish Index, introducing the risk of a lesser urgent response level (non-A responses). Besides the urgency level, the criteria-based dispatch system provided a short text that served as a prenotification of the ambulance personnel.

### Participants

**Fig. 1:**
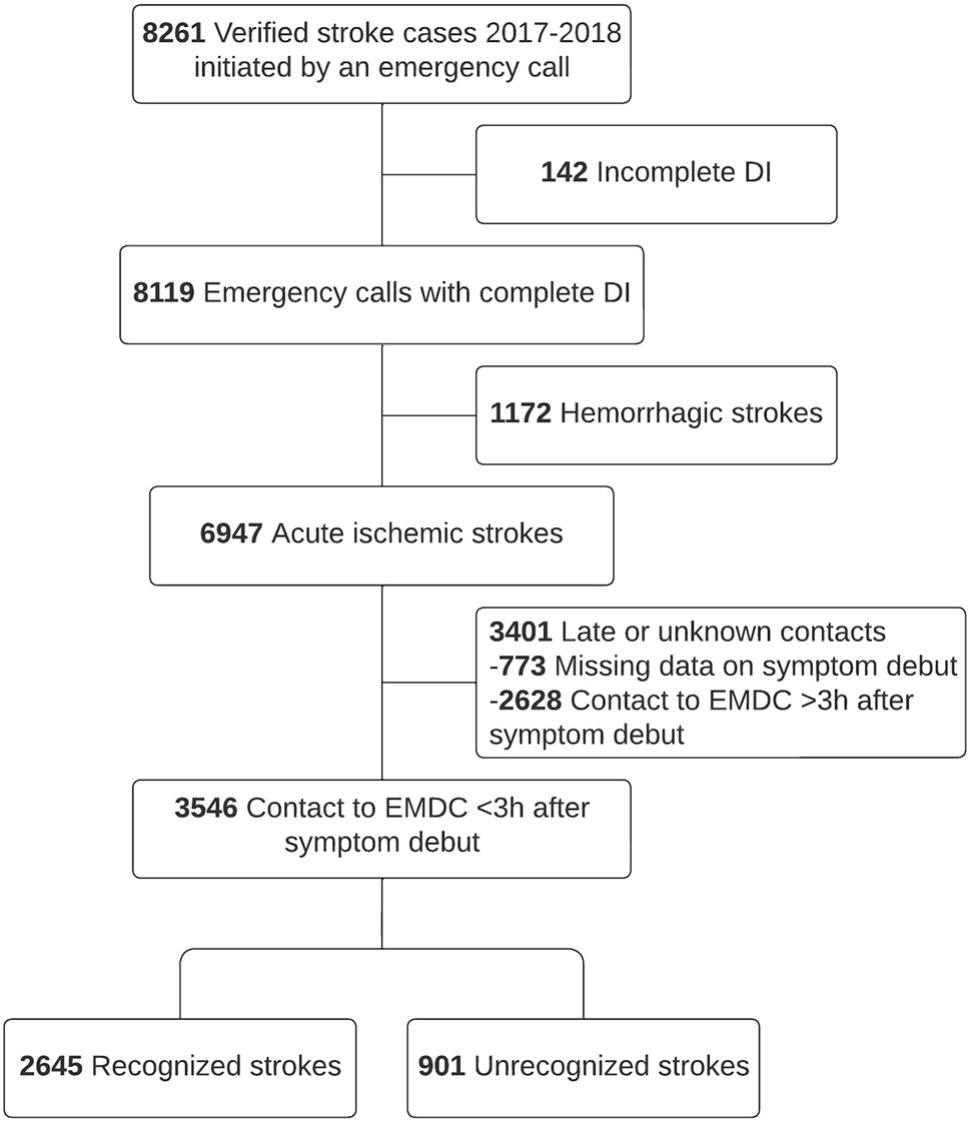
Patient flow chart *DI* danish index (determine EMDC triage), *EMDC* emergency medical dispatch center, recognized/unrecognized refers to the initial identification by the emergence medical service (EMS) dispatcher

The study included all stroke patients diagnosed with AIS in Denmark in 2017 and 2018 for whom the initial healthcare contact was an emergency call made within three hours after symptom debut (Fig. 1). The three-hour time limit was chosen as all patients within this prehospital time window were potentially eligible for acute reperfusion treatment. The three-hour limit was chosen to allow for a realistic transport time and in-hospital evaluation before revascularization within the 4.5-h limit. Stroke diagnoses were established by stroke neurologists according to the World Health Organization stroke definition and registered in the Danish Stroke Registry (DSR).

### Data sources

The DSR is a national clinical quality register holding information about patients diagnosed with stroke or TIA. The DSR was used to identify patients in 2017 and 2018. A sensitivity > 90% of all acute strokes has been reported for the DSR [42]. Information in the database covered final diagnosis, any acute revascularization treatment, patients’ basic characteristics, and their comorbidities, and the National Institute of Health Stroke Scale (NIHSS) for patients receiving acute stroke treatment.

Prehospital data from 2017 to 2018 were stored in databases linked to the computer-aided dispatch systems used by the individual EMDCs. The databases contained time stamps (recordings of the exact time at which various actions had occurred in the prehospital management (e.g., the initial call to the EMDC, ambulance dispatch, ambulance arrival at and departure from the location, and arrival at the hospital)). The databases also contained information about any chosen chapter and subheading in the Danish Index, indicating what symptom/description the EMS dispatcher found to be most fitting and describing the assigned level of urgency [40].

#### Exposure based on EMDC stroke triage

Our study population was divided into two groups depending on whether a stroke was recognized by the EMS dispatcher or not. All patients included in the study were subsequently diagnosed with stroke according to the guidelines.

#### Outcomes

The outcomes were: initial admission at a hospital offering i.v. thrombolysis or EVT, the use of i.v. thrombolysis or EVT, and prehospital delay.

Total prehospital delay accounted for all delays from the initial EMDC emergency call to a stroke admission including all subsegments in the prehospital management. Subsegments of total prehospital delay were: EMDC delay (time from EMDC call to ambulance arrival at the patient’s location/the scene); on-scene time (time from arrival to and departure from the scene); transport to hospital (time from scene departure to arrival at first hospital); and door-in-door-out (time from arrival to and departure from hospital) for patients with temporary admissions before transferal to a stroke admission including i.v. thrombolysis or EVT as indicated.

To calculate the true positive rate (sensitivity) of EMDC stroke triage, we used the diagnosis registered in the DSR as outcome (denominator).

#### Covariates

Sex, age at admission, previous stroke or TIA, and response level (A and B–as C and D was few) were used for the adjusted analysis. Stroke severity measured by the NIHSS (assessed at admission) was additionally used for adjustments for patients receiving acute stroke treatment—the only patients where NIHSS was persistently registered.

### Statistics

Descriptive statistics on basic characteristics, primary admission to hospitals offering revascularization treatment, response levels, and stroke severity (for patients receiving acute stroke treatment) were performed comparing recognized with unrecognized stroke groups. The number of missing variables was low, and imputation was not used. Numbers were presented for all calculations. Differences in stroke management were calculated both as a risk difference (RD) and relative risk (RR) using linear regression and Poisson regression, respectively, both with robust variance and inverse probability of treatment weights (IPTW) for all adjusted analyses.

We calculated the true positive rate (sensitivity) for EMDC stroke triage to identify acute ischemic stroke.

To calculate prehospital delays, we used linear regression with robust variance and IPTW for adjusted analyses. Balanced diagnostics were conducted using the threshold criteria given by Zhang et al. [43]. To calculate a total association, we adjusted for sex, age, previous stroke or TIA, and stroke severity measured by the NIHSS. The NIHSS was assessed at admission before treatment and only for patients receiving either i.v. thrombolysis or EVT. Additionally, to calculate a direct association, the response level A was used for adjustment to show any residual association between EMDC triage and prehospital delay beyond the higher frequency of level-B responses dispatched in the unrecognized stroke group.

All data were analyzed using Stata 15 (StataCorp. College Station, TX: StataCorp LLC.).

## Results

From 2017 to 2018, 8,261 patients had a verified stroke for which an emergency call to the EMDC initiated the healthcare contact (Fig. 1). We excluded 142 patients because of incomplete EMDC stroke triage, 1,172 patients with intracranial hemorrhage, 773 patients with missing data on symptom onset, and 2,628 patients with a contact with the EMDC more than three hours after symptom onset. Patients with AIS and an emergency call to the EMDC within three hours after symptom onset totaled 3546, constituting our study population (Fig. 1). The true positive rate of EMDC stroke triage (sensitivity) for AIS and a symptom duration ≤ 3 h was 74.6% (95% CI 73.1–76.0) – hence, 2645 patients had a stroke that was recognized by the EMS dispatcher and 901 patients had an unrecognized stroke. Recognized and unrecognized only refers to the initial contact with the EMDC – all patients were eventually diagnosed with stroke.

Patients with stroke recognized and unrecognized by the EMS dispatcher were comparable regarding their basic characteristics and comorbidity (Table 1). In addition, the median NIHSS was comparable between the EMDC triage groups among patients treated with i.v. thrombolysis and/or thrombectomy (Table 1). On a subgroup level, median NIHSS differed significantly among EVT-treated patients for recognized versus unrecognized stroke (14 vs. 17) (data not shown).

**Table 1:** Basic characteristics of patients with AIS and an emergency call within three hours of onset

Factor	Unrecognized stroke by the EMDC (all)	Recognized stroke by the EMDC (all)	Unrecognized stroke by EMDC (acute stroke treatment)	Recognized stroke by EMDC (acute stroke treatment)
*N*	901	2645	375	1312
Sex (female)	393 (43.6%)	1111 (42.0%)	158 (42.1%)	526 (40.1%)
Age at symptom debut, median (IQR)	74.1 (63.8, 81.8)	73.3 (64.1, 81.1)	72.6 (60.6, 79.7)	72.2 (62.6, 79.7)
NIHSS (IQR)	n/a	n/a	6 (3, 14)	5 (3, 11)
Living alone	316 (37.5%)	876 (34.7%)	114 (32.0%)	373 (29.4%)
Nursing home	39 (4.6%)	169 (6.6%)	9 (2.5%)	49 (3.8%)
Alcohol overuse	87 (11.8%)	288 (12.8%)	34 (11.0%)	141 (12.6%)
Smoking (non)	299 (41.7%)	885 (39.8%)	126 (42.3%)	426 (38.4%)
Smoking (present)	159 (22.2%)	603 (27.1%)	67 (22.5%)	320 (28.8%)
Smoking (former)	259 (36.1%)	734 (33.0%)	105 (35.2%)	364 (32.8%)
Diabetes	136 (15.2%)	363 (13.8%)	50 (13.5%)	157 (12.1%)
Atrial fibrillation	194 (21.7%)	521 (19.8%)	60 (16.2%)	206 (15.8%)
Acute myocardial infarction	79 (8.9%)	224 (8.6%)	32 (8.7%)	116 (8.9%)
Hypertension	494 (55.9%)	1496 (57.0%)	200 (54.1%)	716 (55.2%)
Previous stroke	206 (23.3%)	724 (27.6%)	76 (20.6%)	304 (23.4%)
Previous transient ischemic attack	61 (7.0%)	251 (9.7%)	24 (6.6%)	115 (8.9%)
Peripheral artery disease	52 (6.0%)	130 (5.0%)	13 (3.5%)	61 (4.7%)

Unrecognized/recognized stroke = strokes that were unrecognized or recognized by the EMS dispatchers, (all) = total group of patients with a contact to the EMDC within three hours after symptom debut, (acute treatment) = subgroup of the total group of patients treated with either i.v. thrombolysis or endovascular treatment

*EMDC* emergency medical dispatch center, *IQR* interquartile range, *i.v.* intravenous, *NIHSS* National Institute of Health Stroke Scale

In the total study population (*n* = 3546), the proportion of patients with an initial admission to a hospital offering acute stroke treatment including i.v. thrombolysis and/or EVT was higher for patients with recognized stroke compared with unrecognized stroke; 85.8% (95% CI 84.4–87.1) versus 74.5% (95% CI 71.5–77.3) yielding an adjusted RD of 11.1% (95% CI 7.8; 14.3) (Table 2). Subsequently, a higher rate of revascularization treatment was seen in the group with recognized stroke; 49.6% (95% CI 47.7; 51.5) versus 41.6% (95% CI 38.4; 44.9) with an adjusted RD of 8.4% (95% CI 4.6; 12.2). For i.v. thrombolysis alone, an even greater difference was seen in favor of recognized stroke; 46.7% (95% CI 44.8–48.7) versus 37.7% (95% CI 34.5–41.0) with an adjusted RD of 9.5% (95% CI 5.7; 13.2) (Table 2). However, the proportion of patients receiving EVT did not differ between the two groups; 10.6% (95% CI 9.4; 11.8) in the recognized group and 10.5% (95% CI 8.6; 12.7) in the unrecognized group (Table 2).

**Table 2:** Prehospital management and treatment for EMDC stroke triage groups (recognized and unrecognized acute ischemic stroke)

Factor	Recognized stroke by EMDC	Unrecognized stroke by EMDC	RD, RR (95% CI)^a^
*N*	2645	901	
Admissions			
Initial admission to a stroke center with i.v. thrombolysis or EVT	2258 (85.8%)	668 (74.5%)	RD = 0.111 (0.078; 0.143) RR = 1.148 (1.101–1.198)
Inter-hospital transfer before i.v. thrombolysis (*n* = 1565)	37 of 1226 (3.0%)	29 of 339 (8.6%)	RD = − 0.054 (− 0.086; − 0.022) RR = 0.364 (0.226; 0.586)
Inter-hospital transfer before EVT (*n* = 366 – 9 missing values)	93 of 274 (33.9%)	37 of 92 (40.2%)	RD = -0.049; (− 0.166; 0.068) RR = 0.869 (0.629; 1.201)
Acute revascularization treatment			
All acute revascularization treatments ^c^	1312 (49.6%)	375 (41.6%)	RD = 0.084 (0.046; 0.122) RR = 1.202 (1.101; 1.313)
i.v. Thrombolysis	1226 (46.7%)	339 (37.7%)	RD = 0.095 (0.057; 0.132) RR = 1.251 (1.138; 1.376)
EVT	280 (10.6%)	95 (10.5%)	RD = 0.002 (− 0.216; 0.025) RR = 1.016 (0.810; 1.275)
Prehospital urgency level (all patients *n* = 3546)			
Response level A	2624 (99.2%)	622 (69.0%)	RD = 0.303 (0.272; 0.334) RR = 1.440 (1.376; 1.506)
Response level B	21 (0.8%)	274 (30.4%)	–
Response level C	0 (0.0%)	2 (0.2%)	–
Response level D–E	0 (0.0%)	3 (0.3%)	–
Total distance, median (IQR)	24.2 (10.5, 50.4)	20.3 (8.1, 43.3)	–
Prehospital urgency level (patients with acute revascularization treatment (*n* = 1687)			
Response level A	1306 of 1312 (99.5%)	290 of 375 (77.3%)	RD = 0.247 (0.196; 0.297)^b^ RR = 1.330 (1.243; 1.422)^b^
Response level B	6 of 1312 (0.5%)	85 of 375 (22.7%)	–

a unrecognized stroke as reference and RD and RR are adjusted for sex, age, and previous stroke or TIA

b additional adjusted for the National Institute of Health Stroke Scale score

c i.v. thrombolysis (*n* = 1565) and thrombectomy (*n* = 375)–253 were treated with both

*CI* confidence interval, *EMDC *emergency medical dispatch center, *EVT* endovascular treatment, *IQR* interquartile range, *NIHSS* national institute of health stroke scale, *RD* risk difference, *RR* relative risk, Unrecognized/recognized stroke strokes that were unrecognized or recognized by the EMS dispatchers

Of the 1,565 patients treated with i.v. thrombolysis, only 66 patients received treatment after inter-hospital transfer. A need for inter-hospital transfer applied to more admissions in the unrecognized stroke group compared with the recognized stroke group; 8.6% (95% CI 5.8–12.1) versus 3.0% (95% CI 2.1–4.1) (Table 2). This higher rate of inter-hospital transfers among patients with unrecognized stroke was seen although these patients arrived later at a hospital not offering i.v. thrombolysis (median 118.0 versus 79.4 min). A need for interhospital transfer before EVT also applied to more admission in the unrecognized stroke group, but the difference was not statistically significant (Table 2).

Patients with stroke recognized by the EMS dispatcher almost exclusively received a level-A response (99.2%); patients with an unrecognized stroke received a level-A response in 69.0%, a level-B response in 30.4%, and a level-C or D-response in 0.6% of all dispatches (Table 2). The same magnitude of difference in receiving a level-A response was seen among patients who received i.v. thrombolysis and/or EVT with an adjusted RD of 24.7% (95% CI 19.6; 29.7) (Table 2).

For all patients (*n* = 3546), the total association (difference in means) between EMDC stroke triage and prehospital delay was − 33.2 min (95% CI − 44.4; − 22.0) in favor of recognized stroke. The total association was adjusted for sex, age at admission, and previous stroke and TIA (Table 3).

**Table 3:** Difference in prehospital delays between EMDC stroke triage groups (recognized and unrecognized acute ischemic stroke)

Description	Difference in minutes & 95% CI	Mean in minutes & 95% CI (reference group)
Prehospital delay (all patients – *n* = 3546)		
Crude unadjusted association with prehospital delay (*n* = 3461)	− 34.4 (− 46.0; – 22.8)	97.9 (86.6; 109.1)
Total association (adjusted^a^) (*n* = 3384)	– 33.2 (– 44.4; – 22.0)	96.3 (85.4; 107.1)
Direct association (adjusted^a^ and additional adjusted for response level) (*n* = 3384)	– 26.8 (– 38.3; – 15.3)	89.7 (78.5; 100.9)
Prehospital delay (patients treated with acute revascularizing treatment – *n* = 1687)		
Crude unadjusted association with prehospital delay (*n* = 1683)	– 14.2 (– 22.9; – 5.4)	72.1 (63.8; 80.4)
Total association (adjusted^b^) (*n* = 1579)	– 12.6 (– 18.9; – 6.3)	69.9 (64.0; 75.8)
Direct association (adjusted^b^ and additional adjusted for response level) (*n* = 1579)	– 9.4 (– 15.4; – 3.5)	66.0 (60.6; 71.4)
Response level association with prehospital delay (all patients – *n* = 3546). (adjusted^a^)		
Prehospital delay (*n* = 3380)	– 44.8 (– 66.9; – 22.6)	112.6 (90.7; 134.4)
EMDC delay (*n* = 3446)	– 10.3 (– 11.9; – 8.6)	19.2 (17.6; 20.8)
Response level association with prehospital delay (treated with acute revascularizing treatment—*n* = 1687). (adjusted^b^)		
Prehospital delay (*n* = 1578)	– 31.6 (– 56.0; – 7.2)	88.3 (64.0; 112.7)
EMDC delay (*n* = 1577)	– 10.8 (– 14.7; – 6.9)	19.8 (15.9; 23.7)

Reference group was unrecognized stroke for all analysis except the response level association with prehospital delay for which response level-B was reference

a adjusted for sex, age at admission, previous stroke and TIA

b additional adjusted for stroke severity as NIHSS (National Institute of Health Stroke Scale)

EMDC emergency medical dispatch center, EMDC delay from EMDC call to ambulance arrival at scene, EVT endovascular treatment. Prehospital delay from EMDC call to arrival at the hospital offering thrombolysis or EVT or department of neurology for non-acute-treated patients, *ref* reference

Among patients who received acute revascularizing treatment (n = 1,687), the EMDC stroke recognition was still associated with reduced prehospital delay (Table 3). The total association was -12.6 min (95% CI − 18.9; − 6.3) and the direct association was − 9.4 min (95% CI − 15.4; − 3.5). The analyses were adjusted for sex, age at admission, previous stroke and TIA and NIHSS and the direct association was also adjusted for ambulance level of urgency (Table 3).

All sub-elements of the prehospital delay contributed to the entire difference in prehospital delay, except for “transport to hospital” (time from departure from scene to arrival at hospital) (Table 4). Additional adjustments for response level (the direct association) removed the difference in delay from emergency call to arrival at the scene (EMDC delay) indicating that the delay was driven by the response level only (Table 4).

**Table 4:** Sub analysis of the difference in prehospital delays between EMDC stroke triage

Description	Difference in minutes & 95% CI	Mean in minutes & 95% CI (reference group)
Sub analysis of the total association (all patients – *n* = 3546) (adjusted^a^)		
EMDC delay (*n* = 3451)	– 3.2 (– 3.9; – 2.5)	12.2 (11.5; 12.9)
On-scene time (*n* = 3443)	– 2.1 (– 2.8; – 1.4)	22.3 (21.7; 23.0)
Transport to hospital (*n* = 3458)	1.49 (0.2; 2.8)	22.7 (21.6; 23.8)
Length of stay at 1st hospital before transfer (door-in-door-out) (*n* = 222–6 missing data points)	– 104.5 (– 181.5; – 27.4)	290.8 (224.9; 356.8)
Sub analysis of the direct association (additional adjusted ^a^for response level) (all patients – *n* = 3546)		
EMDC delay (n = 3451)	– 0.2 (– 0.9; 0.4)	9.9 (9.4; 10.3)
On-scene time (n = 3443)	– 2.3 (– 3.1; – 1.5)	22.4 (21.7; 23.1)
Transport to hospital (*n* = 3458)	1.8 (0.4; 3.3)	22.3 (21.1; 23.6)
Length of stay at 1st hospital before transfer (door-in-door-out) (*n* = 222 – 6 missing data points)	– 108.5 (– 194.0; – 23.1)	295.0 (219.3; 370.6)
Sub analysis of the total association (patients treated with acute revascularizing treatment – *n* = 1687) (adjusted^b^)		
EMDC delay (*n* = 1577)	– 2.1 (– 3.1; – 1.1)	11.2 (10.2; 12.1)
On-scene time (*n* = 1573)	– 3.4 (– 4.5; – 2.2)	22.5 (21.5; 23.6)
Transport to hospital (*n* = 1578)	1.6 (– 0.3; 3.6)	21.5 (19.8; 23.2)
Length of stay at hospital before transfer (door-in-door-out) (191 transferred – 130 before EVT, 66 before i.v. thrombolysis and 5 patients represented in both categories – 14 missing data points)	– 23.5 (– 49.9; 2.9)	116.2 (92.9; 139.5)
Sub analysis of the direct association (additional adjusted ^b^ for response level) (treated with acute revascularizing treatment – n = 1687)		
EMDC delay (*n* = 1577)	0.1 (– 0.7; 1.0)	9.2 (8.5; 9.9)
On-scene time (*n* = 1573)	– 3.3 (– 4.7; – 1.9)	22.4 (21.2; 23.6)
Transport to hospital (*n* = 1578)	0.3 (– 2.0; 2.6)	22.2 (20.3; 24.1)
Length of stay at hospital before transfer (door-in-door-out) (191 transferred – 130 before EVT, 66 before i.v. thrombolysis and 5 patients represented in both categories – 14 missing data points)	– 20.5 (– 44.0; 3.0)	113.2 (93.2; 133.2)

Reference group was unrecognized stroke for all analysis except the response level association with prehospital delay for which response level-B was reference

a = adjusted for sex, age at admission, previous stroke and TIA

b = additional adjusted for stroke severity as NIHSS (National Institute of Health Stroke Scale)

*EMDC* emergency medical dispatch center, *EMDC* delay = from EMDC call to ambulance arrival at scene, *EVT* endovascular treatment

prehospital delay = from EMDC call to arrival at the hospital offering thrombolysis or EVT or department of neurology for non-acute-treated patients, ref = reference transport to hospital = departure from scene to arrival at the hospital offering thrombolysis

The association between the response level and total prehospital delay was − 44.8 min (95% CI − 66.9; − 22.6); and EMDC delay was − 10.3 min (95% CI − 11.9; − 8.6) – using response level B as reference (Table 3).

## Discussion

In this national cohort study, we found that EMS dispatchers identified AIS with a high sensitivity when symptom onset had occurred within three hours of the patient contacting the EMDC. AIS identified by the EMS dispatchers was associated with both a greater probability of admission to a hospital offering revascularization treatment (85.8% versus 74.5%) and subsequently a greater rate of acute revascularization treatment (49.6% versus 41.6%).

EMS dispatcher stroke recognition was associated with a substantial reduction in prehospital delay compared with non-recognition, both in the overall study population (− 33.2 min) and in the time-critical patient group treated with i.v. thrombolysis and/or EVT (− 12.6 min). A direct association persisted with reduced delay for recognized stroke for all patients (− 26.8 min) and patients treated with i.v. thrombolysis and/or EVT (− 9.4 min) even when adjusting for the relatively frequent use of response level-B in the unrecognized stroke group.

Every minute counts when patients await thrombolysis [44]. Therefore, decreasing delay from symptom onset to thrombolysis remains the main focus in stroke management. We found a substantial reduction in prehospital delay for patients with recognized stroke even when restricting the focus to patients with AIS potentially eligible for acute revascularization within 4.5 h.

Reduced prehospital delay for stroke recognized by EMS dispatchers was also shown in a study by Caceres et al. (49.8 reduced to 41.8 min) [23]. They defined patients with stroke as those patients in whom the EMS providers suspected stroke in the prehospital environment. The study thus lacked a gold standard outcome measure. EMS dispatchers’ and EMS providers’ performance ability to identify patients with stroke is known to be similar with sensitivities of up to 60–70% [45], and the delay reductions reported by Caceres et al. therefore apply to identified patients with a mix of diagnoses (including stroke mimics).

Unlike us, Oostema et al. found no difference in total prehospital delay between EMDC stroke triage groups [25]. The frequency of the highest level of urgency (level A) was in favor of recognized stroke (87.3 versus 72.6%), like in our study, whereas the rate of thrombolysis was not. The discrepancy regarding prehospital delay and thrombolysis rate found in our study versus the findings in the study by Oostema et al. may possibly be explained by a difference in study setups. Oostema et al. included patients from only two centers, both offering thrombolysis. The risk of being admitted to a hospital without thrombolysis therefore did not exist in their study, and those admissions contributed to the lower rate of thrombolysis and the prehospital delay in our study. Disregarding, that no difference was found in overall prehospital delay, the on-scene time differed significantly and reached the same level as reported by us [25]. A difference in on-scene-time that have also been shown by Abbas et al. and Heemskerk et al. favoring recognized stroke [30, 46].

The additional on-scene time for unrecognized stroke found in our study did not depend on the response level and could be due to lacking EMS provider prenotification. If the EMS dispatcher does not recognize a stroke case, EMS providers may have to spend more time at the scene, evaluating the patient more extensively, before a suspicion of a stroke may come to mind.

Prehospital stroke identification is a complex process involving both the EMS dispatcher and the EMS providers. Any prehospital delay due to inability to recognize the condition is hence influenced by the performance of both groups. On the other hand, EMDC delay is not influenced by the EMS providers because it occurs before they arrive. The reduced EMDC delay for the recognized stroke group was mediated by the response level; hence, the response-level-adjusted analysis (direct association) showed no difference in EMDC delay between the groups (Table 4). This corresponds well with the standard operating procedure that all response levels other than level A may be dispatched with an allowed delay. It also demonstrates that a level-A response is equally fast, disregarding the type of indexation (positive or negative stroke triage). Response level B almost exclusively occurred in the unrecognized stroke group. Comparing response level B with response level A revealed a more than twofold increased EMDC delay, and an additional prehospital delay beyond this initial delay.

We reported the performance of EMDC triage and its association with stroke management in a group of patients with AIS where acute stroke treatment is well established and in a time-restricted manner so that all included patients could reach a hospital offering i.v. thrombolysis and/or EVT despite their initial geographical location—a group of patients that has not been reported on previously [26].

Only 66 of the patients with an EMDC contact occurring more than three hours after their initial call received i.v. thrombolysis in contrast to 1,565 patients in the group in which the initial healthcare contact occurred within the three-hour limit. This emphasizes that the time limit used in this study very precisely targets patients eligible for acute stroke treatment. By including patients from all geographical locations in Denmark and from two consecutive years based on a well-validated quality data source (the Danish Stroke Register), we believe that our results are generalizable to other stroke care systems – i.e., that our results have a high external validity.

As a limitation, a risk exists that we have underestimated the association between EMDC stroke triage and prehospital delay and acute revascularization treatment. A patient admitted to an ED, without acute stroke admission, due to a missing stroke identification may have lost the time for reperfusion therapy due to a late stroke identification. In that case, the patient would probably have remained at the first hospital to which they were admitted without inter-hospital transfer because acute treatment was considered timewise out of reach. In our study setup, this group would not contribute to measurable delay. For that reason, the results regarding prehospital delays must be considered in conjunction with the overall rate of i.v. thrombolysis associated with EMDC stroke triage (46.7% versus 37.7%) as a substantial group of patients with unrecognized stroke received no acute treatment. The importance of initial correct admission for receiving i.v. thrombolysis is illustrated by the fact that only a few patients (66 of 603) received i.v. thrombolysis after inter-hospital transfer. Despite having EDs without i.v. thrombolysis in Denmark, 19% of all patients with acute ischemic stroke (AIS) were treated with i.v. thrombolysis within the 4.5-h time limit in 2017 [47]; 22%, in 2018 [48]. Treatment with EVT for large-vessel occlusion was performed in 4.1% in 2017 [47]; 6.0%, in 2018 [48].

Another limitation to the study is a risk of residual unmeasured confounding because the prehospital stroke management consists of several steps of which we have only focused on the initial emergency call to the EMDC. A difference between the EMDC stroke triage groups could exist with unrecognized patients having an atypical presentation both at the initial emergency call and in later management. However, based on the available data, the groups seem to be fairy comparable (Table 1) where the small difference in previous stroke or TIA have been statistically balanced by the IPTW.

## Conclusion

In summary, among a group of AIS patients all of whom had a timely, relevant contact to healthcare, a stroke recognized by the EMS dispatcher was associated with a reduced prehospital delay, a higher chance of primary admission to a hospital offering acute stroke treatment, and subsequently a higher chance of receiving acute revascularization treatment. Even among patients reaching acute stroke treatment in time, the group of patients with stroke recognized by EMDC experienced a substantial and clinically relevant reduced prehospital delay.

A focus on EMS dispatcher stroke recognition may reduce prehospital delay further.

## Data availability statement

The datasets are available through the corresponding author upon reasonable request and permissions according to Danish legislation.
